# Visual Attention and Color Cues for 6D Pose Estimation on Occluded Scenarios Using RGB-D Data

**DOI:** 10.3390/s21238090

**Published:** 2021-12-03

**Authors:** Joel Vidal, Chyi-Yeu Lin, Robert Martí

**Affiliations:** 1 Computer Vision and Robotics Institute, University of Girona, 17003 Girona, Spain; robert.marti@udg.edu; 2 Department of Mechanical Engineering, National Taiwan University of Science and Technology, Taipei 106, Taiwan; jerrylin@mail.ntust.edu.tw; 3 Taiwan Building Technology Center, National Taiwan University of Science and Technology, Taipei 106, Taiwan; 4 Center for Cyber-Physical System Innovation, National Taiwan University of Science and Technology, Taipei 106, Taiwan

**Keywords:** 6D pose estimation, 3D object recognition, RGB-D data, scene understanding, computer vision, model-based vision

## Abstract

Recently, 6D pose estimation methods have shown robust performance on highly cluttered scenes and different illumination conditions. However, occlusions are still challenging, with recognition rates decreasing to less than 10% for half-visible objects in some datasets. In this paper, we propose to use top-down visual attention and color cues to boost performance of a state-of-the-art method on occluded scenarios. More specifically, color information is employed to detect potential points in the scene, improve feature-matching, and compute more precise fitting scores. The proposed method is evaluated on the Linemod occluded (LM-O), TUD light (TUD-L), Tejani (IC-MI) and Doumanoglou (IC-BIN) datasets, as part of the SiSo BOP benchmark, which includes challenging highly occluded cases, illumination changing scenarios, and multiple instances. The method is analyzed and discussed for different parameters, color spaces and metrics. The presented results show the validity of the proposed approach and their robustness against illumination changes and multiple instance scenarios, specially boosting the performance on relatively high occluded cases. The proposed solution provides an absolute improvement of up to 30% for levels of occlusion between 40% to 50%, outperforming other approaches with a best overall recall of 71% for the LM-O, 92% for TUD-L, 99.3% for IC-MI and 97.5% for IC-BIN.

## 1. Introduction

The precise determination of an object’s location in a given scene is a key capability to archive flexible interaction and manipulation to perform autonomous operations [1,2]. Traditionally, most computer vision research efforts have been centered on the detection and classification of objects in monocular images; however, only a few studies have focused on explicitly solving the full six degree-of-freedom problem. In this line, most approaches face the problem from a 2D point of view, rather than inferring the precise rotation and position of the objects in the 3D space, commonly known as the 6D pose estimation problem. Within the last decade, with the rise of machine learning, novel monocular methods appeared showing increasing levels of robustness [3]. However, most of these approaches were still limited to the understanding of the scene in terms of object classification, segmentation, and bounding box detection. Only recently, methods based on deep learning [4,5,6,7] have shown promising results in solving the problem from a 6D pose estimation perspective.

In another direction, methods based on three-dimensional scene data have been the best solutions to robustly solve the 6D pose estimation problem for different types of objects and complex scenarios. A vast variety of solutions based on local features [8], such as PFH [9], SHOT [10], PPF [11] and related methods [12,13], global features, such as VHP [9] or ESF [14], template-matching approaches like Linemod [15], and also machine learning methods based on deep learning [16,17] or random forests [18] have been proposed. The existing wide range of traditional and more recent approaches and techniques evaluated under different platforms, procedures and datasets drew a rather complex picture of the state-of-the-art. In order to obtain a clearer picture of the state of the field, Hodan et al. [19] presented an extensive benchmark for 6D pose estimation of a single instance of a single object (SiSo) task, where different challenging existing and new datasets were collected under a standardized evaluation procedure. Initially, 15 different methods were evaluated and, since then, the 6D pose estimation of a varying number of instances of a varying number of objects (ViVo) has been introduced and more methods have been tested. Among these solutions, methods based on the Point Pair Features (PPF) voting approach [11] have shown some of the best performances. These approaches combine benefits of a global object definition and a local matching approach by matching the object’s and scene’s surface data using feature quantization, a voting-based corresponding grouping and clustering process. Only recently, deep learning methods are reaching higher levels of accuracy, although they require a tremendous amount of data and long training procedures, compared to the single training 3D model of PPF. Among all methods, our previous PPF-based depth-only solution presented in [20] obtained the best overall result in the BOP Challenge SiSo task in 2017, the best overall result in 2019, and best depth-only result in 2020 for the ViVo task. This solution proposes a six-step pipeline that focuses on extracting more discriminate information from the surface data and uses additional refinements and improvements to boost its performance. Despite the good performance of those top scoring methods against clutter and illumination changes, results show that occluded scenarios still remain challenging. In particular, for the SiSo task, results obtained on the Linemod occluded (LM-O) dataset [21,22] show a clear weakness of state-of-the-art methods against occluded cases, decreasing the overall recognition results from 91% to 62% when compared to the non-occluded version Linemod (LM) dataset. In a more detailed analysis presented in [19], recall scores related to the visible fraction of the target object show that more recent methods performance decrease to less than 10% recognition rates when occlusion levels reach 50% of the object.

In this paper, we propose to incorporate color information and visual attention principles to boost the performance of a state-of-the-art pose estimation method for highly occluded scenarios. Specifically, we propose to improve the method presented in [20] by using color information to guide the attention of the method to potential scene zones and improve surface matching.

Visual attention is an important biological mechanism based on selecting subsets of the world information to perform faster and more efficient scene understanding. Inspired by the understanding of the human visual system and the development of more efficient intelligent applications, visual attention has been an important research topic in both neuroscience and computer vision fields. Either based on bottom-up or top-down architectures [23], different computer vision methods for visual attention have been presented behind the ideas of salient maps [24], object-based attention [25], and saliency feature vectors [26]. Potapova et al. [27] presented a survey of visual attention from a 3D point of view, analyzing 3D visual attention for both human and robot vision. Their work reviews the most important attention computational models presented, from the widely used contrast-based saliency models [24] to the recently proposed Convolutional Neural Network (CNN) learning approaches [28]. On this line, most research done on visual attention has focused on biologically inspired bottom-up attentional mechanisms. For most solutions, the generalized idea of salient feature identification is applied to optimize the application of the limited computational resources to the most attractive elements, regardless of the final task or prior knowledge. This pathway, however, does not completely match the requirements of occluded scenarios, where target objects may not necessary be prominent or highly distinguishable attention elements in the scene. Hence, top-down mechanisms, where previously known features are identified as salient scene points for potential targets, are considered more suitable. Therefore, following this direction, we propose to integrate a top-down attention mechanism to the method presented in [20] by using color cues as prior knowledge of the object.

Although studies suggest that color contributes to biological object recognition [29], traditionally, color information has been scarcely applied to computer vision recognition approaches. While most methods rely on shape and texture information [30], only a few cases have considered color information as a prominent feature. Although this situation has been abruptly reversed with the rising of artificial neural networks approaches, for which color information is usually considered, only few traditional model-based solutions have relied on color information for object detection and recognition, such as color SIFT features [31] for 2D vision or CSHOT [32] and VCSH [33] for 3D vision. For the PPF voting approaches, Drost et al. [34] proposed a multimodal variant of the original method [11], defining pairs of oriented 3D points and 2D gradient edges. The proposed method showed a noticeable improvement on performance while showing robustness to light, having the main drawback of a big impact on the runtime performance [19]. In a different direction, Choi et al. [35] proposed to extend the PPF to 10 dimensions, including color information from both points underneath surface. Although showing positive results on some datasets, more recent results presented in the Ref. [36] suggest that the inclusion of the color on the PPF may provide, for some cases, higher precision results, but lower recognition rates. The deterioration of the recognition rates can be attributed to the subjugation of the geometric information to the color information, disregarding valid geometrical matches for non-matching color cases produced by illumination changes, modeling artifacts or different sensors characteristics. Arguably, this undesired behavior can be considered the main reason why historically few traditional model-based methods have relied on color data. In general, the mathematical modeling of the color information, including their dependency on the sensor technology, is much more complex and unstable than other available features, like gradients or geometrical features. In a different direction, we propose to use the color information only as a cue of a correct matching on the top of the existing geometrical approach based on PPF. The main idea is to use the color information as a weighting factor to provide more relevance to color consistent cases and help to distinguish between geometrically ambiguous cases.

Therefore, we present a novel solution based on visual attention and color cues to boost performance of a state-of-the-art method on highly occluded cases. First, we propose to use a top-down attention mechanism to focus the method on those parts of the scene that potentially belong to the object. Secondly, we propose to use color information as a weighting factor to improve the geometrical matching of the method. The proposed solutions have been evaluated on the SiSo task for different parameters, color spaces and metrics against the state-of-the-art benchmark occluded LM-O dataset. Results show that the proposed method obtains a very significant improvement against occluded cases, increasing recognition rates for relatively low visible objects, outperforming the other solutions. In addition, the proposed solution has been tested under different illumination conditions on the TUD-L dataset, obtaining better performance than previous methods and showing the robustness of the proposed approach to illumination changes. Finally, the method robustness has been also tested for cases with multiple instances for two different datasets, IC-MI and IC-BIN, showing robustness against scenes with a high number of repeated color patterns of the target object.

## 2. Method

We propose to integrate the attention-based approach and the color cue weighting solution in a state-of-the-art PPF voting approach. Specifically, the method of [20] is extended by using color information to identify a set of salient points that will guide the attention of the pose estimation algorithm, decreasing the complexity of the global matching problem while increasing the chances of obtaining a positive result. In addition, the color information is used as a weighting factor for the matching of point pairs and re-scoring step to increase the relevance of the color consistence of geometrical data.

### 2.1. The Point Pair Features Voting Approach

We base the proposed solution on a Point Pair Features (PPF) voting approach, extending with color the depth-only method presented in [20]. The PPF voting approach, first introduced by Drost et al. [11], is a feature-based method that globally defines an object as the set of pairs of the oriented points that defines its surface, allowing a local matching of the object in a given scene by only matching a subset of these pairs.

The pairs are individually matched by using 4D features that encode the distance between the pair of points and the difference between their normal angles. More specifically, for a set of model points M, a PPF is defined between a reference and second points $m_{r},m_{s}\in M$ with their respective normal vectors $n_{m_{r}}$ and $n_{m_{s}}$, as shown in Equation (1):

(1) $$F\left(m_{r},m_{s}\right)=[\;||d||,∠\left(n_{m_{r}},d\right),∠\left(n_{m_{s}},d\right),∠\left(n_{m_{r}},n_{m_{s}}\right){\;]}^{T},$$

where, $d=m_{s}-m_{r}$ and ∠(a,b) is the angle between the vectors a and b. As the number of all possible point pairs combinations is determined by a square factor, the method proposes to reduce the overall number of points by downsampling both scene and model data with respect to the object diameter. Similar point pairs, i.e., that define similar surface features, are grouped together on a hash table by quantizing the feature space. This table defines the object model as a mapping from the quantized PPF to the set of their corresponding model point pairs. Later, during the scene matching, this table is used to determine scene-model point pair correspondences, which are grouped in geometrically consistence 6D poses representing potential candidate poses for the object in the scene. This corresponding grouping relays on the fact that pairs sharing the same reference point can be efficiently grouped on a 2D space in a Hough transform manner. Specifically, for a given scene point belonging to the object model surface, a candidate pose is represented by a local coordinate (LC), which is defined by two parameters; a corresponding model point and the rotation around their aligned normals. The method defines a two-dimensional accumulator for each scene reference point where each cell represents a LC. Then, all pairs defined from this reference point are matched against the model and each scene-model pair correspondence is used to define a LC that casts a vote in the accumulator. The most voted LC defines a potential candidate pose. Finally, similar candidate poses obtained from different scene reference points are joined together using a clustering approach.

Starting from here, the method presented in [20] further extends this idea by proposing a set of novel and improved steps on an integrated local pipeline. First, the preprocessing part is improved, proposing a resolution independent process for normal estimation and introducing two novel downsampling steps to optimize the method performance by filtering non-discriminative surface data. These two steps check the normal variation between neighbouring points, clustering and filtering cases that have similar normal information. During matching, the method uses a more efficient kd-tree structure for neighbouring search and includes two additional improvements to tackle problems derived from the quantization and the over-representation of similar scene features. In addition, a novel threshold is introduced after correspondence grouping to discard low supported poses from the accumulator. A complete-linkage clustering approach is also proposed to improve the original clustering step, which can join similar poses more robustly. Another relevant improvement is the introduction of an accurate solution to recompute the object fitting score by counting the number of model’s points matching the scene. This process employs the model’s render view refined by an efficient variant of the Iterative Closest Point (ICP) method. Finally, two different verification steps are included to discard false positive cases which do not consistently fit the scene surface in terms of visibility context and geometrical edges. Overall, the method showed a significant improvement with respect to [11] for varying types of objects and scene cases, showing to outperform the other methods for different types of datasets under clutter and occlusion. Further details about the method can be found in the Ref. [20].

### 2.2. Attention-Based Matching Using Color Cues

**Figure 1:**
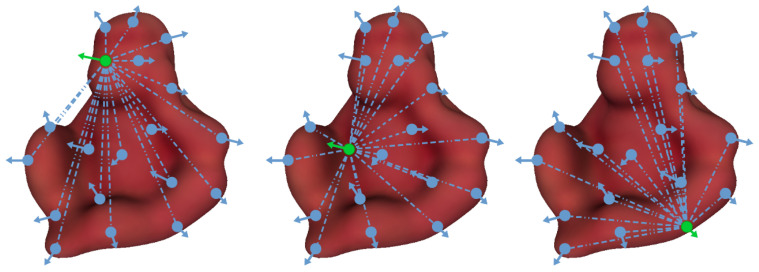
Illustration of the Point Pair Feature voting approach showing how a 3D object model is globally defined as a set of locally matched oriented points pairs (in blue) from each reference point (in green).

The PPF voting approach is characterized for describing the whole object model as a set of oriented pairs from each of its points, as shown in Figure 1. As explained before, the matching process relies on finding for each scene reference point the best LC, i.e., corresponding model point and rotation angle, that better fits the object model in the scene, i.e., most voted cell in the accumulator. Indeed, only scene reference points that truly belong to the object model will have a matching corresponding model point, and thus a correct LC. Therefore, all the other scene points will only add superfluous cases, i.e., wrong hypothesis, that will increase processing time and the likelihood of a final mismatching. From this point of view, the right selection of these reference points is an important element of the method performance which has been underestimated so far. In fact, up to now most available approaches propose to use a blind-search approach, using all scene points [35,37] or a fixed random fraction of them, usually one-fifth [11,20].

If we consider a rather more intuitive human perception approach, an object could be more efficiently found by focusing attention on zones of the scene that contains elements or features which resemble the ones of the object and can potentially be part of it. However, there is a number of reasons, i.e., occlusion, illumination changes, imperfections, for which those zones could not be properly identified and therefore, the whole scene should be searched. In that case, it seems reasonable to search the scene at a regular intervals related to the object size. Hence, we propose to combine two different strategies: (1) to focus the matching attention on parts of the scene that are similar to the object; and (2) to search the whole scene at constant intervals. Following this reasoning, and taking advantage of the PPF voting approach nature of matching an object from a single reference point, we propose to center the attention of these matching points on scene points that have similar color to the object as well as selected points distributed homogeneously at fixed spaced intervals. Therefore, the matching attention will be focused on salient points that are selected based on their relevance in the image (i.e., their color prominence) as well as their spatial distribution.

In order to identify the scene points that have color similar to the object and can potentially belong to an object part, we propose to check the color similarity between each of the scene points and the object model. As a single object can have multiple colors on its surface and in different amounts, we only consider those scene points for which their color is found multiple times in the model surface. Therefore, for each scene point, we propose to use a color metric to search all model points with similar color and only use those scene points with a minimum number of matching color model points, which are more likely part of the object. Specifically, for a given scene point s∈S, the set of similar color model points is defined by Equation (2),

(2) $$C\left(s\right)=\{\;m:d_{c}\left(s,m\right)<\alpha ,\;m\in M\}$$

where $d_{c}\left(\right)$ is a color distance metric between two points and α is a threshold bounding the similarity level. Then, the set of reference points used to center the method attention is defined by the cardinality of color matching points as defined by Equation (3),

(3) R(S)={s:|C(s)|≥β,s∈S}

where β is a threshold bounding the minimum number of color matches for a scene point to be considered.

In another direction, a voxel-grid structure is defined to divide the scene at fixed regular distance intervals on the three dimensions. These divisions are used to determine an homogeneous distributed set of potential points on the 3D space. In practice we propose to divide the scene using a voxel size of 10% of the object diameter and to use the nearest point to the voxel’s center as a reference point.

**Figure 2:**
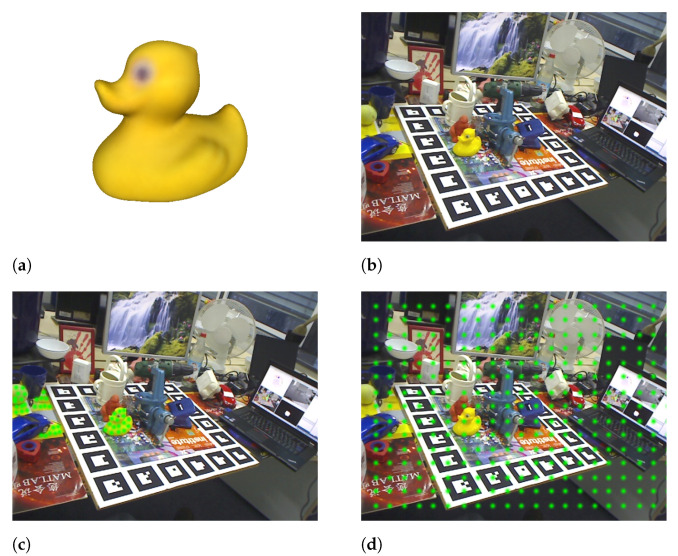
Representation of the attention-based reference points selection methodology. (**a**) Duck object model; (**b**) a scene containing the Duck object; (**c**) the scene with the selected reference points (green) that are potentially part of the Duck object. (**d**) The scene with a 2D representation of the reference points (green) distributed at regular intervals.

Figure 2 shows a representation of the two different proposed reference points selection strategies for the Duck object.

### 2.3. Color-Weighted PPF Matching

In addition to raising attention on potential scene zones, the object model color information can be used to improve the matching process. Choi and Christensen [35,38] proposed a straightforward approach to use the color information underneath each point pair using the HSV color space to define 10 dimensional features, which include both the geometrical and color data. This solution, however, subordinates the 3D geometrical information to the quality of the color information, and vice versa. This subordination implies the requirement of high quality color models and scene data. Otherwise, the solution can dramatically decrease the method performance on low-quality color scenarios produced by the discrepancy and distortion introduced by different sensor properties, illuminations, and the model creation process. We propose a different solution in which color information is used as a weighting factor for geometric data, rewarding those feature correspondences that are consistent with the scene in terms of both geometrical and color information. In this direction, a weight value is applied for each LC on the accumulator to increase the value of those poses supported by color consistent point pairs. The weight value for a given scene-model corresponding point pairs, $s_{r},s_{s}\in S$ and $m_{r},m_{s}\in S$, is defined by Equation (4),

(4) $$W_{\mathrm{pp}}\left(s_{r},s_{s},m_{r},m_{s}\right)=1+W_{c}\left(s_{r},m_{r}\right)\cdot W_{c}\left(s_{s},m_{s}\right)$$

and Equation (5),

(5) $$W_{c}\left(s,m\right)=\left\{\begin{array}{cc} \omega , & d_{c}\left(s,m\right)<\alpha ,\\ 0, & \mathrm{otherwise}, \end{array}\right.$$

where ω is a scalar factor that relates the value of the color information with respect to geometrical data. Notice that the multiplication factor links the consistency of each point of the pair and the added unit accounts for the basic value of the geometrical matching.

As described earlier, the method rescores the clustered candidate poses to obtain a better fitting value to select the best candidate hypothesis. Therefore, the proposed color weighting only affects the corresponding grouping step and color information will not be taken into account after rescoring. Nevertheless, the color information can also be considered to improve rescoring and compute a better fitting score. In this direction, we propose a novel improved rescoring approach which takes into consideration both geometrical and color data. Following the rescoring formula proposed in the Ref. [20], the fitting score is obtained by summing the model points that have a scene nearest neighbour within a threshold. In this work we propose a more refined solution for which the score value of each object’s point is computed by adding the inlier maximum distance plus the additive inverse of the point’s distance, i.e., the Euclidean distance between the object point and its nearest scene point. In this way, inliers that are further away from the surface provide lower scores. Then, to consider color information, this geometric score is multiplied by one plus the color matching weight, in a similar way to the weighted matching of Equation (4). Specifically, for a given pose P which transforms the model M to the scene S, the score is computed as defined by the Equation (6),

(6) $$\begin{array}{cc} \; & S_{\mathrm{color}}\left(P\right)=\\ \; & \sum_{m\in M}\left\{\begin{array}{cc} (th-||\mathrm{Pm}-s_{\mathrm{nn}}\left||\right)\cdot \left(1+W_{c}\left(s_{\mathrm{nn}},m\right)\right), & ||\mathrm{Pm}-s_{\mathrm{nn}}\left|\right|<th,\\ 0, & \mathrm{otherwise}, \end{array}\right. \end{array}$$

where,

(7) $$s_{\mathrm{nn}}=\underset{s\in S}{arg min}\{||\mathrm{Pm}-s||\}$$

represents the nearest neighbour from an object model point to the surface and *th* represents the inliers maximum distance threshold, which is set to half of the downsamping voxel size, as in the Ref. [20].

### 2.4. Color Models and Distance

Color information can be affected by scene conditions (i.e., illumination and shadows), sensor properties (e.g., exposition time, white balance, resolution), and object modeling processes. In this direction, we have taken into account several combinations of most used different color models and metrics to determine the most robust solution.

First, we consider the RGB color space [39], as the most standardized solution. We propose to use the $L_{2}$ norm as defined by Equation (8),

(8) $$\begin{array}{cc} L_{2}\left(s,m\right) & =\sqrt{{\left(R_{s}-R_{m}\right)}^{2}+{\left(G_{s}-G_{m}\right)}^{2}+{\left(B_{s}-B_{m}\right)}^{2}}. \end{array}$$

We also consider the HSV/HSL [39] spaces, due to their known illumination invariant properties. Similarly to RGB, we propose to use a variant of the $L_{2}$ metric, which takes into consideration the particularities of the Hue dimension of both spaces, and this metric $L_{2}\mathrm{Hue}$ is defined by Equation (9)

(9) $$\begin{array}{cc} \; & L_{2}\mathrm{Hue}\left(s,m\right)=\sqrt{\Delta H^{2}+\Delta S^{2}+\Delta L^{2}}\\ \; & \Delta H=\min(\mathrm{abs}\left(H_{s}-H_{m}\right),1-\mathrm{abs}\left(H_{s}-H_{m}\right))\\ \; & \Delta S=S_{s}-S_{m}\\ \; & \Delta L=L_{s}-L_{m} \end{array}$$

Finally, we have also considered the CIELAB color space [39,40], as a perceptually uniform space with respect to human vision. This color space provides a device-independent color model with respect to a defined white point. Although conceived and mostly used in the industry, this complex color space has also been tested before for other 3D computer vision methods [32]. In this case, the CIE94 $\Delta E^{*}$ distance metric is used as a trade-off between accuracy and speed, defined by Equation (10),

(10) $$\begin{array}{cc} \; & \mathrm{CIE}94\left(s,m\right)={\left[{\left(\frac{\Delta L^{*}}{K_{L}S_{L}}\right)}^{2}+{\left(\frac{\Delta C_{ab}^{*}}{K_{C}S_{C}}\right)}^{2}+{\left(\frac{\Delta H_{ab}^{*}}{K_{H}S_{H}}\right)}^{2}\right]}^{\frac{1}{2}},\\ \; & \Delta L^{*}=L_{m}^{*}-L_{s}^{*}\\ \; & C_{m}^{*}=\sqrt{a_{m}^{{*}^{2}}+b_{m}^{{*}^{2}}}\\ \; & C_{s}^{*}=\sqrt{a_{s}^{{*}^{2}}+b_{s}^{{*}^{2}}}\\ \; & \Delta C_{ab}^{*}=C_{m}^{*}-C_{s}^{*}\\ \; & \Delta H_{ab}^{*}=\sqrt{\Delta a^{{*}^{2}}+\Delta b^{{*}^{2}}-\Delta C_{ab}^{{*}^{2}}}\\ \; & \Delta a^{*}=a_{m}^{*}-a_{s}^{*}\\ \; & \Delta b^{*}=b_{m}^{*}-b_{s}^{*}\\ \; & S_{L}=1\\ \; & S_{C}=1+0.045C_{m}^{*}\\ \; & S_{H}=1+0.015C_{m}^{*} \end{array}$$

where the model point is considered as the standard reference and the parameters are set like graphic arts applications under reference conditions with $K_{L}=K_{C}=K_{H}=1$. Notice that the LAB color space transformation has been done by using the X, Y, and Z tristimulus reference values for a perfect reflecting diffuser, using the standard A illuminant (incandescent lamp) and 2° observer (CIE 1931). The reader can refer to the Refs. [39,40] for more details about this color space and its metrics.

### 2.5. Precomputing Color Weights

It can be observed that the color weight between a scene and model point, i.e., Equation (5), is computed for each point pair correspondence during matching, i.e., Equation (4), and for each model point during rescoring, i.e., Equation (6). Therefore, the weight value for the same scene-model combination is required multiple times for both cases, significantly increasing the method’s running time. This problem can be easily solved by precomputing the weight for every scene-model point combination in a lookup table. In this way, the given weight for any scene-model point combination can be found by accessing the lookup table in a constant time. As during this weight precomputing process all scene-model points will be checked, we propose to also determine the attention reference points simultaneously. To obtain further efficiency, for the $L_{2}$ and $L_{2}\mathrm{Hue}$ metrics, we propose to create a kd-tree structure with the object model color information that can help to efficiently retrieve the model points with similar color information.

## 3. Results

We analyse and compare the performance of the proposed method using different datasets for the SiSo task as part of the standardised Benchmark for 6D Object Pose Estimation (BOP) of rigid objects [19]. As the main aim of the method is to improve pose estimation in hihgly occluded escenarios, we performed the main evaluation using the Linemod occluded dataset. In addition, and to show its robustness to illumination variations and multiple instances, we propose to also include the TUD light, the Tejani et al. (IC-MI) and the Doumanoglou et al. (IC-BIN) datasets. First, the performance of the different proposed color spaces and metrics for occlusion cases are evaluated and its parameters (i.e., alpha, beta, omega) defined for the best overall configuration using the Linemod occluded dataset. Second, the proposed method is compared against the other state-of-the-art approaches using the same dataset. Third, the method robustness under illumination changes is evaluated using the TUD light dataset. Finally, the performance on multiple instances is evaluated for different scenarios using the IC-MI and IC-BIN datasets.

### 3.1. Datasets and Evaluation Metric

The proposed method has been analysed and evaluated on the Linemod occluded dataset (LM-O) [15,22] for occlusion performance, on the TUD light dataset (TUD-L) for robustness to illumination changes and on the Tejani et al. dataset (IC-MI) [18] and Doumanoglou et al. dataset (IC-BIN) [41] for multiple instance robustness, all as part of the SiSo task of the BOP benchmark [19].

**Figure 3:**
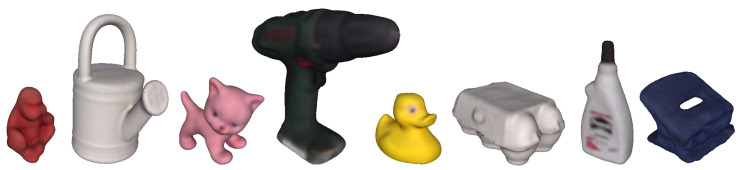
Objects models used on the LM-O dataset. From left to right objects are 1-Ape, 5-Can, 6-Cat, 8-Driller, 9-Duck, 10-Eggbox, 11-Glue, and 12-Holepuncher.

**Figure 4:**
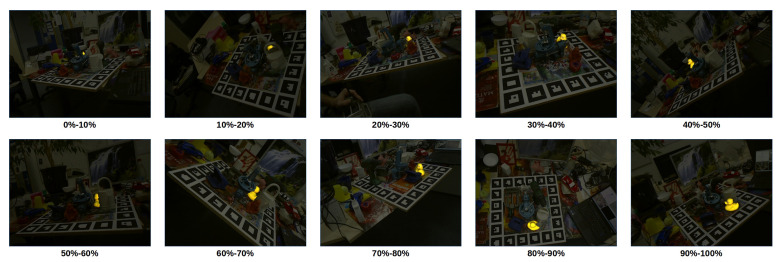
Ground truths for different levels of visibility of the Duck object in the LM-O dataset.

The LM-O dataset was created by [22] from the background scenes of the well-known and already mastered Linemod dataset [15] to define a more challenging scenario with highly occluded cases. Later, the dataset was refined with fixed ground truths and included as part of BOP [19], a benchmark for 6D pose estimation of rigid objects from a single RGB-D image. Specifically, the LM-O dataset features eight different objects, shown in Figure 3, with different colors, shapes and sizes laying on multiple poses and with different levels of visibility on highly cluttered scenes. Overall, the main challenge of the dataset relies on the high level of occlusion of some of its scenes, having target cases with visibility levels from 100% up to 0%, although, based on the benchmark evaluation criteria, poses under 10% are not considered. As a reference, to visualize the difficulty of the dataset, Figure 4 shows ground truths for different visibility levels for one of its objects, known as Duck.

**Figure 5:**
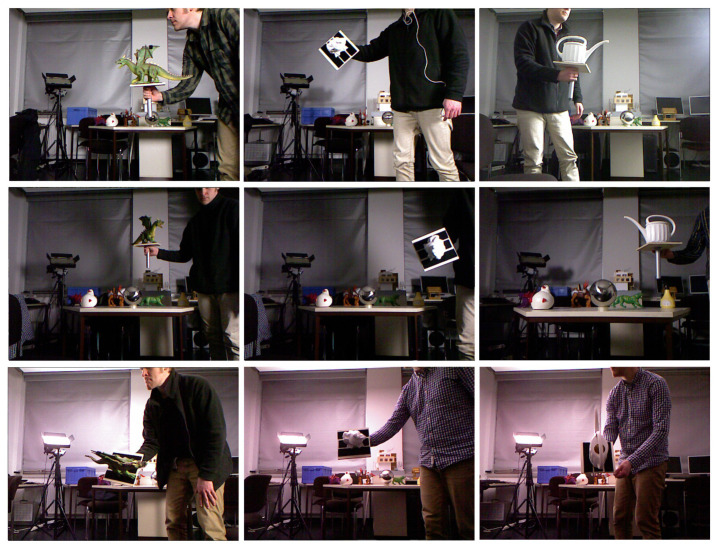
Examples of test images with different light configurations for the three models of the TUD-L dataset. From column left to right objects are 1-Dragon, 2-Frog, 3-Watering pot.

The TUD-L is a light-focused dataset, with limited clutter and occlusion, that features different challenging illumination conditions. The dataset was specifically created as part of the BOP benchmark and includes three randomly moving objects, namely Dragon, Frog and Watering pot, under eight different illumination conditions. Some examples images are shown in Figure 5.

**Figure 6:**
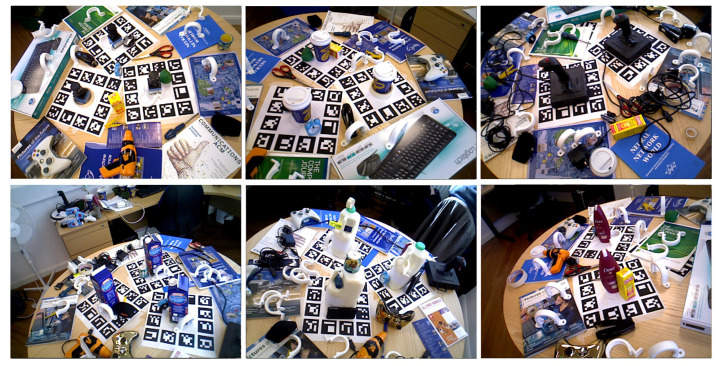
Examples of test images for each object of the IC-MI dataset. From left to right and from top to bottom, the results are: 1-Camera, 2-Coffee cup, 3-Joystick, 4-Juice carton, 5-Milk, and 6-Shampoo.

The IC-MI is a multiple object instance dataset with clutter and slight occlusion. The dataset includes six household objects, namely, Coffee cup, Shampoo, Joystick, Camera, Juice carton, and Milk (see Figure 6).

**Figure 7:**
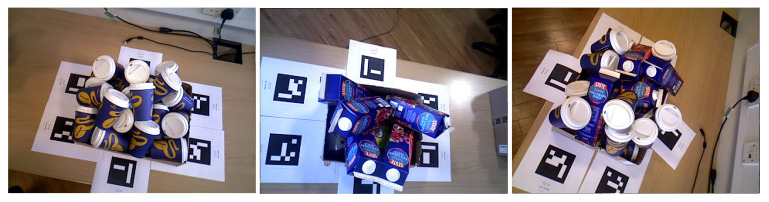
Examples of test images for each scene of the IC-BIN dataset using the objects 2-Coffee cup and 4-Juice carton from IC-MI.

Finally, the IC-BIN features two objects from IC-MI, namely, the Coffee Cup and Juice Carton, on a classic multiple-instance random bin-picking scenario (see Figure 7).

For all datasets, each object includes textured-mapped 3D object models and training images of synthetic scenes. For our method, only the textured-mapped 3D objects are required. For evaluation, we have followed the BOP benchmark criteria specified in the Ref. [19], using the Visible Surface Discrepancy (VSD) evaluation metric with a misalignment tolerance τ=20 mm and correctness threshold θ=0.3.

### 3.2. LM-O: Performance and Parameter Configuration

In this section, the performance of the method is evaluated for the different color spaces and normalized metrics discussed to determine the best overall configuration using the Linemod occluded dataset. First, the alpha, beta, and omega parameters are determined to achieve the best overall performance. Alpha (α), Equations (2) and (5), defines the color similarity. Beta (β), Equation (3), defines the minimum number of color matching points. Finally, omega (ω), Equation (5), defines the relative weight of color data. Although the beta and omega parameters are expected to be invariant for different configurations, the optimal alpha parameter is related to the metric value and depends on the color space and metric configuration. Therefore, the beta and omega values are initially determined heuristically and set to 10 and 5, respectively, and later rechecked for the optimal alpha values. Based on this, a detailed analysis of the alpha parameter for the different proposed color model and normalized metric configurations is conducted. Specifically, the method is evaluated as the obtained overall recall on the LM-O dataset for different alpha values for the RGB color space with the $L_{2}$ metric, i.e., Equation (8), the HSV and HSL color spaces with the $L_{2}\mathrm{Hue}$ metric, i.e., Equation (9), and finally the CIELAB color space with the CIE94 metric, i.e., Equation (10). Then, for reference, these results have been compared with the ones obtained for the depth-only original solution of the Ref. [20].

**Figure 8:**
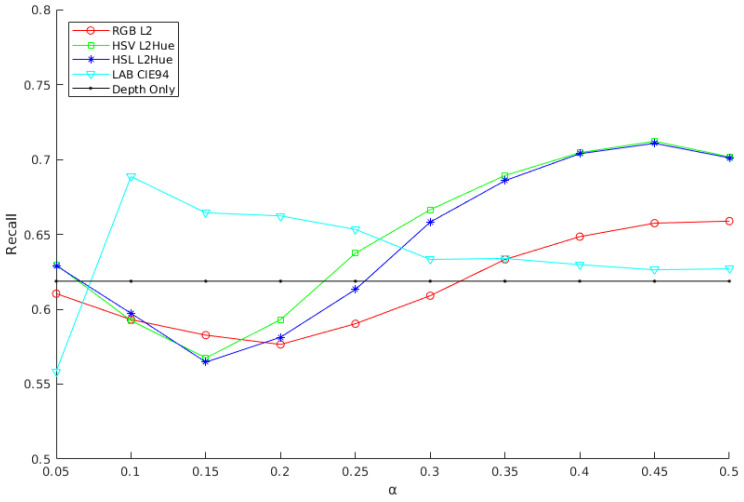
Evaluation of different color spaces and metrics with respect to the alpha value.

Overall, this test will help to determine the best alpha value for each color space and metric combination. Results presented in Figure 8 show a consistent sinusoidal-like behavior for different alpha values with positives result for values bigger than 0.32 for most cases besides CIELAB, reaching a great improvement in performance for all color configurations. We attribute the CIELAB different shape but mostly positive behaviour to their unique human vision related nature. All tested cases improve the results obtained with the original depth-only method [20], obtaining the best recognition rates with alphas 0.5, 0.45, 0.45 and 0.1, for the RGB $L_{2}$, HSV $L_{2}\mathrm{Hue}$, HSL $L_{2}\mathrm{Hue}$ and CIELAB CIE94 cases, respectively. Specifically, HSV, HSL and CIELAB obtain better results than RGB, attributable to their better illumination modeling. On its turn, HSV and HSL obtain relatively better results than CIELAB, although the latest one shows a rather more stable performance with respect to alpha. The worse performance observed with respect to the depth-only for small alpha values can be explained by the fact that small thresholds are prone to capture ambiguous color information that produce a larger weight for color mismatches.

We should notice that the performance of these color spaces and metrics is evaluated under an unknown and uncontrolled color-related sensor, illumination and object modeling characteristics, which do not follow a human-like color modeling. Finally, HSV shows very similar behaviour with HSL, although the former one obtains slightly better results. Overall, the the HSV solution reaches the highest recognition rate, with a recall value of 71.21% with an alpha of 0.45, obtaining a very significant improvement with respect to the 61.87% obtained by the depth-only solution [20], proving the value of the added color information. As mentioned before, parameters beta and omega were tested again for these alpha values, obtaining optimal results for the initial choices, showing an expected stable behaviour for all different configurations.

**Figure 9:**
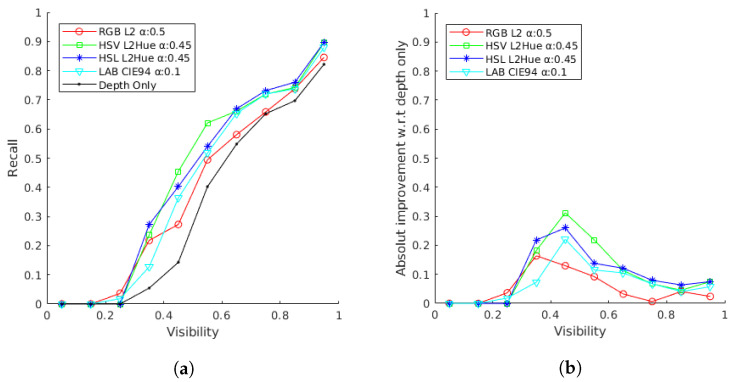
Results obtained using different color and metric cases for the best alpha with respect to the object visibility level. (**a**) Overall recognition rate; (**b**) Absolute improvement rate with respect to [20].

Second, the performance of the different solutions using the best alpha values in terms of the object visibility is evaluated. Specifically, the recall rate for the best obtained alpha has been plot with respect to the visibility percentage of the objects. Recognition rates obtained by the depth-only method have been also included as a reference of the obtained improvement. The obtained results are presented in Figure 9 in terms of recall and absolute recall improvement with respect to the depth-only method. The results show a consistent improvement for all color configurations and all visibility levels higher than 20%, demonstrating the stability of the color information improvement. In particular, the results show the added value of the proposed method on the targeted occluded cases, specially, rising the performance of cases with an occlusion level lower than 60%, with improvements of about 20%, 30% and 20% for object with a 30–40%, 40–50%, 50–60% visibility, respectively. Following the previous results, the best overall performance for all the visibility spectrum is obtained on the HSV space, although noticeable recognition rates of 1.8% for LAB and 3.6% for RGB are obtained for a very low 20–30% of visibility.

**Figure 10:**
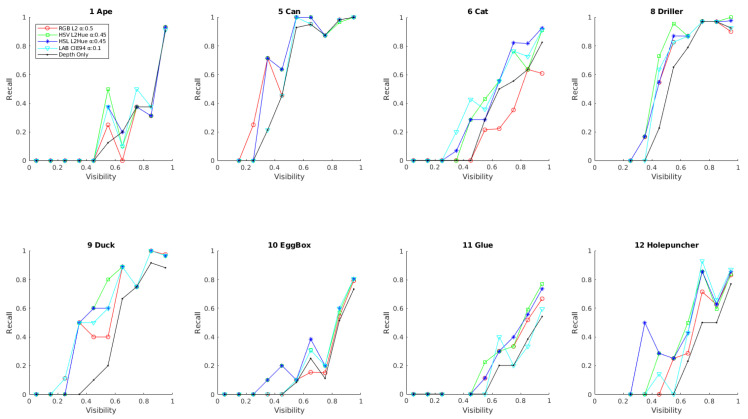
Recall value for each LM-O dataset object using different color space and metric combinations for the best alpha with respect to the object visibility rate.

Finally, a detailed picture of the robustness against occlusion obtained for each different object is presented in Figure 10. As can be seen and following previous results, most obtained improvements with respect to depth-only are localized on relatively low levels of visibility, showing a very noticeable and consistent improvement of robustness against higher occluded cases. Analysing the results in more detail, the objects Ape, Can, Driller and Eggbox show an improvement mainly on visibility levels lower than 80%. On the same line, objects Cat, Duck, Glue and Holepuncher shows improvement for all levels of visibility. Overall, Can and Driller are the most robust objects, reaching recognition rates of near 100% for cases with near 50% occlusion. The improved robustness against occlusion can be specially seen on the objects Can and Duck, which reach recognition rates of 71% and 50% for half-visible objects, respectively, representing a tremendous improvement with respect to the depth-only performance of 21% and 0%.

### 3.3. LM-O: State-of-the-Art Comparison

**Figure 11:**
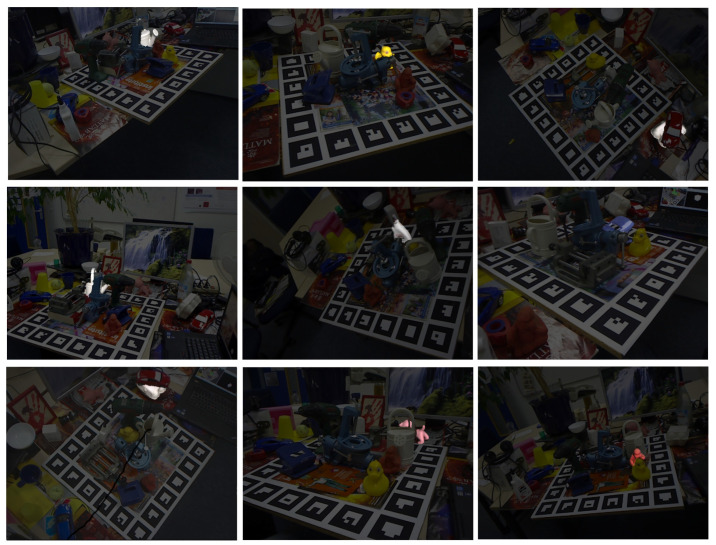
Examples of results obtained with the proposed method. From left to right and from top to bottom, the results are (visibility): Can (39%), Duck (39%), Eggbox (34%), Can(36%), Glue (69%), Holepuncher (48%), Eggbox (50%), Cat (49%) and Ape (60%).

After determining the method’s best parameters and configuration, the proposed solution is compared against the other top scoring state-of-the-art methods for SiSo presented in BOP [19], results presented in the Ref. [20], and the recent deep learning works of Mercier et al. [42], Mitash et al. [43], and Tong et al. [17]. This last work also investigated occlusion using the same dataset. Results are presented in Table 1. As can be seen, the proposed method outperforms all other methods (overall mean of 70 compared to 62 for the second best method) and for 6 out of 8 objects. The challenge of the occlusion problem and the novel benefits introduced by the proposed approach can be observed comparing the results obtained by the other approaches (including deep-learning-based methods) which have been able to obtain an incremental improvement from 51% to 62% in a 10 years period, while the proposed approach jumps to 70%. Examples of successful object pose estimation with a large degree of occlusion for various objects using our method are shown in Figure 11.

The proposed method outperforms all other PPF methods (Drost-10-edge, Drost-10, Vidal-18a and Vidal-18b) for all objects. The inclusion of visual attention principles and color information provides a clear improvement with respect to the previous depth-only work Vidal-18b [20], contrasting with the slightly lower performance obtained by the inclusion of gradient information of Drost-10-edge with respect to Drost-10. Specifically, the proposed method improve 9 points with respect to the previous work in Vidal-18b. The largest improvement has been obtained for object 11 moving from 34% obtained by [20] to 50%. Additionally, objects 8 and 9 show an important improvement of 13 and 12 points, respectively. Relating these results back to Figure 9, it can be observed that some improvements are attributed to all the visible domain, i.e., object 11, although most improvements are obtained for the low visibility cases, i.e., objects 8 and 9.

**Figure 12:**
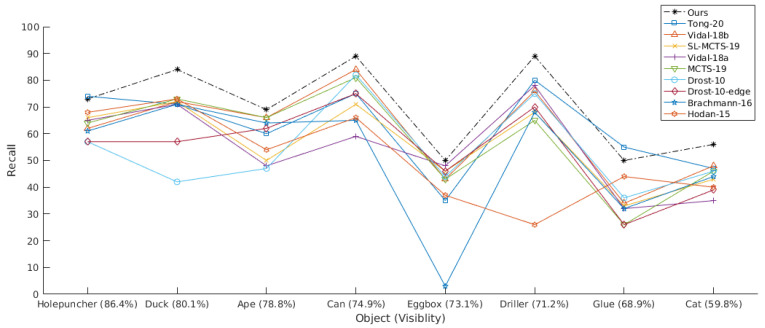
Recall scores (%) for each LM-O object with respect to the object’s mean visibility rate.

Continuing the analysis in terms of visibility, Figure 12 plots the obtained results sorted by the object’s mean visibility rate. As can be seen, the proposed method clearly outperforms all previous state-of-the-art methods for most levels of visibility. Specifically, the plot shows a very noticeable overall improvement with respect to the second best method, the deep learning approach of Tong-20 [17]. The figure also shows how the proposed method improves occlusion cases with respect to deep-only, in special on lower visible objects like Driller and Cat. In addition, based on results of Figure 10, we can conclude that more highly visible objects, such as Duck, have also been mostly improved on low-level visibility rates, further validating the benefits of the proposed solutions for occlusion cases.

### 3.4. TUD-L: Robustness under Illumination Changes

In this section, the proposed method is tested for the TUD light dataset, also part of BOP [19], which includes several different illumination scenes for three different objects. The evaluation has been done following the same metric and same set of parameters as in the previous dataset and compared against the same methods when possible.

As shown in Table 2, the proposed method’s overall results also outperform all the other solutions, showing not only stable performance on illumination changes but also better performance overall for the TUD-L dataset. In this direction, these results under different illumination scenarios shows the robustness of the proposed color cues, used here as a weighing factor, to provide additional performance from color information without decreasing the value of the geometrical matching. A more detailed analysis per object shows clear improvement for object 1 and almost similar or slightly lower performance for objects 2 and 3. This behaviour may be explained by the relevance of the color information of the objects within the scene, as object 1 has prominent colors different from the background, while objects 2 and 3 are much less relevant, as can be seen in Figure 5. Therefore, although the usage of color information shows better performance and robustness to illumination changes for most cases, as expected, its benefits are mostly limited to scenes where color is a meaningful feature.

### 3.5. IC-MI/IC-BIN: Performance on Multiple Instances

Finally, the performance of the method was evaluated for datasets IC-MI and IC-BIN, which includes multiple instance of the target objects. The evaluation on this datasets has also followed the same metric and same parameters as previous cases and compared against the same methods when possible. For IC-MI, which includes multiple instance of a slightly occluded object in highly cluttered scenes, results can be seen in Table 3.

For IC-BIN, which includes a large number of instances in the common random bin-picking configuration for objects 2 and 4 of IC-MI, results are shown in Table 4.

As can be seen, the proposed method outperforms the other solutions for the IC-MI dataset, obtaining the best average score of 99.3% and the same score than deep-only for the random bin-picking setup, IC-BIN, with an average score of 97.5%. Overall, this shows that even in multiple-instance datasets with repeated color patterns, the method performance does not degrade, improving even in cases with lower number of instances compared to using depth-only information.

## 4. Conclusions and Future Work

A novel solution based on visual attention and color cues for improving robustness against occlusion for 6D pose estimation using Point Pair Features voting approach has been presented. The proposed method incorporates color information at different steps: first to identify potential scene points belonging to the object in order to focus the pose estimation method. Secondly, the method uses the color information to weigh the feature-matching and re-scoring step, providing more weight to those points matching both geometry and color. The method has been analyzed on different parameters, color spaces and metrics, showing a better performance for all tested color spaces for the SiSo task on the widely used LM-O dataset. The best result has been obtained with the HSV color space and L2 metric, alpha 0.45, beta 10 and omega 5, showing the benefits of including color cues obtaining an average recall of 70%. Compared to the original PPF-based method without color information, the proposed method obtains an improvement of 9%, which is specially focused in low occlusion levels between 30% to 70%. Compared to the state-of-the-art, the proposed method outperforms all approaches by at least 8% including comparison to current machine learning (deep learning)-based methods. The method’s robustness to illumination changes has been evaluated on the TUD-L dataset, showing stable behavior and obtaining an overall better performance compared to the other approaches, with an improvement limited to the cases with meaningful color information. Finally, the proposed solution has also shown robustness under repeated color patterns when tested against a moderate and high number of multiple instance of the same object on the IC-MI and IC-BIN datasets.

Future work will focus on four main directions. First, study how the presented color solutions can improve other well-known problems faced by object recognition approaches, especially distinguishing objects from identical or similar shapes. Secondly, future work will also focus on investigating richer features based on color and texture patterns that could potentially improve the robustness and results of the method. Third, we will also study more complex color models based on the idea of weighted color for surface features. Finally, we will adapt the current SiSo problem to the slightly different ViVo task, where multiple instance and multiple objects are considered simultaneously.

## Figures and Tables

**Table 1:** Recall scores (%) for the LM-O dataset [15,22] as part of the BOP benchmark [19] using the VSD metric with τ=20 mm and θ=0.3. The recall score for each individual object, the average recall, the standard deviation and the overall recall for all objects are reported. Objects are numerated as specified in [19] and shown in Figure 3.

Method	1	5	6	8	9	10	11	12	Avg.	Stdev	All
Ours - HSV $L_{2}\mathrm{Hue}$	**69**	**89**	**56**	**89**	**84**	**50**	50	73	**70**	17	**71**
Tong-20 [17]	60	75	47	80	71	35	**55**	**74**	62	16	-
Vidal-18b [20]	66	84	48	76	72	43	34	62	61	17	62
Mercier-MS-ICP-19 [42]	-	-	-	-	-	-	-	-	-	-	62
SL-MCTS-19 [43]	50	71	43	68	72	46	33	66	57	15	60
Vidal-18a [44]	66	81	46	65	73	43	26	64	58	18	59
MCTS-19 [43]	48	59	35	78	71	48	32	65	55	17	58
Drost-10-edge [45]	47	82	46	75	42	44	36	57	54	17	55
Drost-10 [11,45]	62	75	39	70	57	46	26	57	54	16	55
Mercier-MS-19 [42]	-	-	-	-	-	-	-	-	-	-	55
Brachmann-16 [46]	64	65	44	68	71	3	32	61	51	24	52
Hodan-15 [47]	54	66	40	26	73	37	44	68	51	17	51

**Table 2:** Recall scores (%) for the TUD-Light dataset as part of the BOP benchmark [19] using the VSD metric with τ=20 mm and θ=0.3. The recall score for each individual object, the average recall and the standard deviation for all objects are reported. Objects are numerated as specified in [19].

Method	1	2	3	Avg.	Stdev
Ours - HSV $L_{2}\mathrm{Hue}$	**92**	94	91	**92**	2
Vidal-18b [20]	88	93	**92**	91	3
Vidal-18a [44]	79	88	74	80	7
Drost-10-edge [45]	85	88	90	87	3
Drost-10 [11,45]	73	90	74	79	10
Brachmann-16 [46]	81	**95**	91	89	7
Hodan-15 [47]	27	63	48	46	18

**Table 3:** Recall scores (%) for the IC-MI dataset as part of the BOP benchmark [19] using the VSD metric with τ=20 mm and θ=0.3. The recall score for each individual object, the average recall and the standard deviation for all objects are reported. Objects are numerated as specified in [19].

Method	1	2	3	4	5	6	Avg.	Stdev
Ours - HSV $L_{2}\mathrm{Hue}$	98	100	100	100	100	98	99.3	1.0
Vidal-18b [20]	94	100	100	100	100	98	98.7	2.4
Vidal-18a [44]	80	100	100	98	100	94	95.3	7.9
Hodan-15 [47]	100	100	100	74	98	100	95.3	10.5
Drost-10 [11,45]	76	100	98	100	96	96	94.3	9.2
Drost-10-edge [45]	78	100	100	100	90	96	94.0	8.8
Brachmann-16 [46]	42	98	70	88	64	78	73.3	19.6

**Table 4:** Recall scores (%) for the IC-BIN dataset as part of the BOP benchmark [19] using the VSD metric with τ=20 mm and θ=0.3. The recall score for each individual object and the average recall for all objects are reported. Objects are numerated as specified in the Ref. [19].

Method	2	4	Avg.
Ours - HSV $L_{2}\mathrm{Hue}$	100	95	97.5
Vidal-18b [20]	100	95	97.5
Vidal-18a [44]	100	93	96.5
Drost-10-edge [45]	100	94	92.0
Hodan-15 [47]	100	81	90.5
Drost-10 [11,45]	100	74	87.0
Brachmann-16 [46]	84	29	56.5
